# Morphological Stability of Intracranial Aneurysms and the Contour Neurovascular System: A Longitudinal Analysis

**DOI:** 10.1007/s13239-026-00847-9

**Published:** 2026-07-13

**Authors:** Lena Iam Spitz, Jana Korte, Franziska Gaidzik, Naomi Larsen, Daniel Behme, Bernhard Preim, Sylvia Saalfeld

**Affiliations:** 1 Department of Simulation and Graphics, Otto-von-Guericke University, Magdeburg, Germany; 2 Research campus STIMULATE, Magdeburg, Germany; 3 Laboratory of Fluid Dynamics and Technical Flows, Otto-von-Guericke University, Magdeburg, Germany; 4 Department of Radiology and Neuroradiology, University Medical Center Schleswig-Holstein, Campus Kiel, Germany; 5 University Clinic for Neuroradiology, Otto-von-Guericke University, Magdeburg, Germany; 6 Department of Medical Informatics, University Hospital Schleswig-Holstein, Campus Kiel, Germany

**Keywords:** Intracranial aneurysm, Morphological analysis, Contour neurovascular system, Wide-necked bifurcation aneurysm

## Abstract

**Purpose:**

The Contour Neurovascular System (CNS) is a novel intrasaccular flow disruptor for the endovascular treatment of wide-necked aneurysms. While more longitudinal studies are needed to fully assess its efficacy, initial study results over the past few years have been promising. However, no studies have yet assessed the CNS’s robustness to deformation over time, and few have assessed its effect on the morphology of intracranial aneurysms.

**Methods:**

Thirteen IAs were retrospectively analyzed using co-registered 3D meshes from serial imaging (2–5 scans per patient, up to 26 months of follow-up). Morphological parameters of the ostium and CNS were compared across defined time points. Intra-patient variance was also assessed through scans taken on the same day, and morphology was correlated with treatment outcome.

**Results:**

Significant postprocedural changes were observed in the ostium’s ellipticity index (EI; Δ = −0.07 ±0.06, p = 0.01, d = −1.18) and neck circularity index (NCI; Δ = +0.04 ±0.04, p = 0.04, d = 0.90). No statistically significant long-term changes were detected for either the ostium or CNS. Most variations were within intra-patient variance, and many measured deformations were below imaging resolution (0.16−0.47 mm). No correlation was found between recurrence and morphology.

**Conclusion:**

Only immediate postprocedural shape changes of the ostium were significant. No measurable long-term deformation of the CNS or ostium was observed. The CNS appears morphologically stable, though larger, high-resolution studies are needed to confirm these findings.

## Introduction

The Contour Neurovascular System (CNS; Stryker, USA) is a novel intrasaccular flow disruptor (IFD) for treatment of wide-necked bifurcation aneurysms, which are associated with relatively high recurrence rates [1]. The CNS is a dual-layered mesh device made from 144 nitinol wires that unfold into a cup-like shape, sitting in the neck of intracranial aneurysms (IAs) to disrupt blood flow into the aneurysm sac.

Longitudinal studies about the CNS are ongoing. Multicenter reviews and studies cover between 131 and 661 ruptured and unruptured IAs and report adequate occlusion rates between 84.21 and 93%, with thromboembolic complications in 6% to 8.53% cases and less than 1% subarachnoid complications [2–7].

The CNS’s promising success rates are comparable to those of other IFDs [1, 3, 4], however, it is considered easier to deploy, as it adapts to the neck of the IA, and device sizing depends on neck and IA width rather than height [8]. The CNS leads to a larger reduction of blood flow into the IA sac with lower flow reduction in parent artery branches than other flow diverters, according to an in-silico study [9]. Other in-silico and in-vitro studies found the inflow of blood into the IA sac to be effectively reduced after CNS deployment, with evidence pointing towards CNS neck coverage and positioning affecting the rate of reduction [10, 11].

Few studies have investigated the influence of IA morphology on CNS success compared to other IFDs [12]. One study found no correlation with pre-treatment IA morphology and outcome [4]. A preliminary study with 7 aneurysms found a shrinkage of the IA neck after CNS deployment of approximately 0.5 $\frac{mm}{year}$, but no other conclusive shape change [13]. To date, no study has assessed the robustness of the CNS implant morphology to deformation. Device deformation of over 50% of pre-deployment parameters can occur in other IFDs and stents and needs to be monitored carefully as it may affect device efficacy [14–16].

This work presents a comprehensive longitudinal deformation analysis of

- the IA neck and ostium after contour deployment over time
- the CNS implant morphology

as well as comparing the results to intra-patient variance and investigating correlation of IA morphology and recurrence.

## Materials and Methods

### Data

**Fig. 1:**
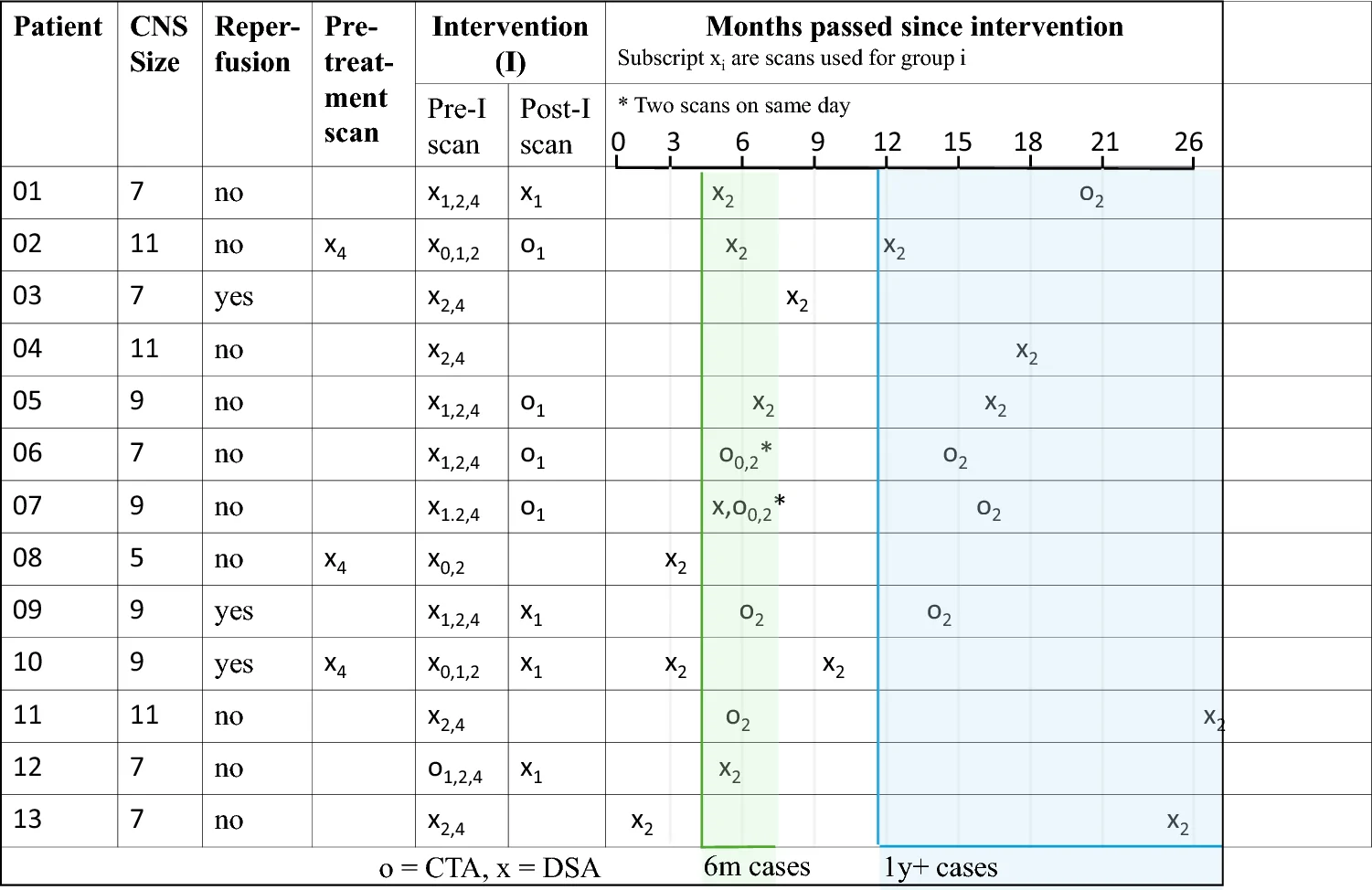
List of cases, their CNS size and whether they have a recurrence, along with scans taken and which group they were assigned for deformation analysis. x marks DSA and o marks CTA scans. The “i” in x_i_ denotes group. * marks dates on which two scans were taken. Group explanation to be found in Sec. Results.

We retrospectively analyzed 13 IAs treated with the CNS from two German hospitals in accordance with the ethical standards of the national research committee. Each IA was scanned between 2 and 5 times. This resulted in a total of 48 scans (see Fig. 1).

### Imaging

Each IA was scanned at least once before and once after CNS deployment, with scans being either 3D rotational DSA or 3D CTA scans. Imaging modality was not fully consistent across all longitudinal timepoints, as follow-up imaging depended on clinical routine and scanner availability at the participating centers. Examinations were performed on biplane angiography systems (Allura Xper FD 20/10, Philips, The Netherlands, or Axiom Artis Q, Siemens Healthcare, Germany), where resolution ranged from $384\times 384\times 384$ to $512\times 512\times 355$, and isotropic voxel size ranged from 0.16 to 0.47 mm after reconstruction.

### Longitudinal Data

Most IAs had multiple follow-up scans after treatment, and extra scans taken during CNS deployment, amounting to a total of 48 scans (see Fig. 1). Pre-treatment scans were done for three IAs. All thirteen IAs had a pre-interventional scan taken right before deployment, and eight had a postprocedural scan right after deployment. Those that did not have a postprocedural scan taken had at least one follow-up scan. Nine IAs had a second follow-up, and two had a third.

The average time between the first and last scan was 16 months, with a minimum of six and a maximum of 26 months.

**Fig. 2:**
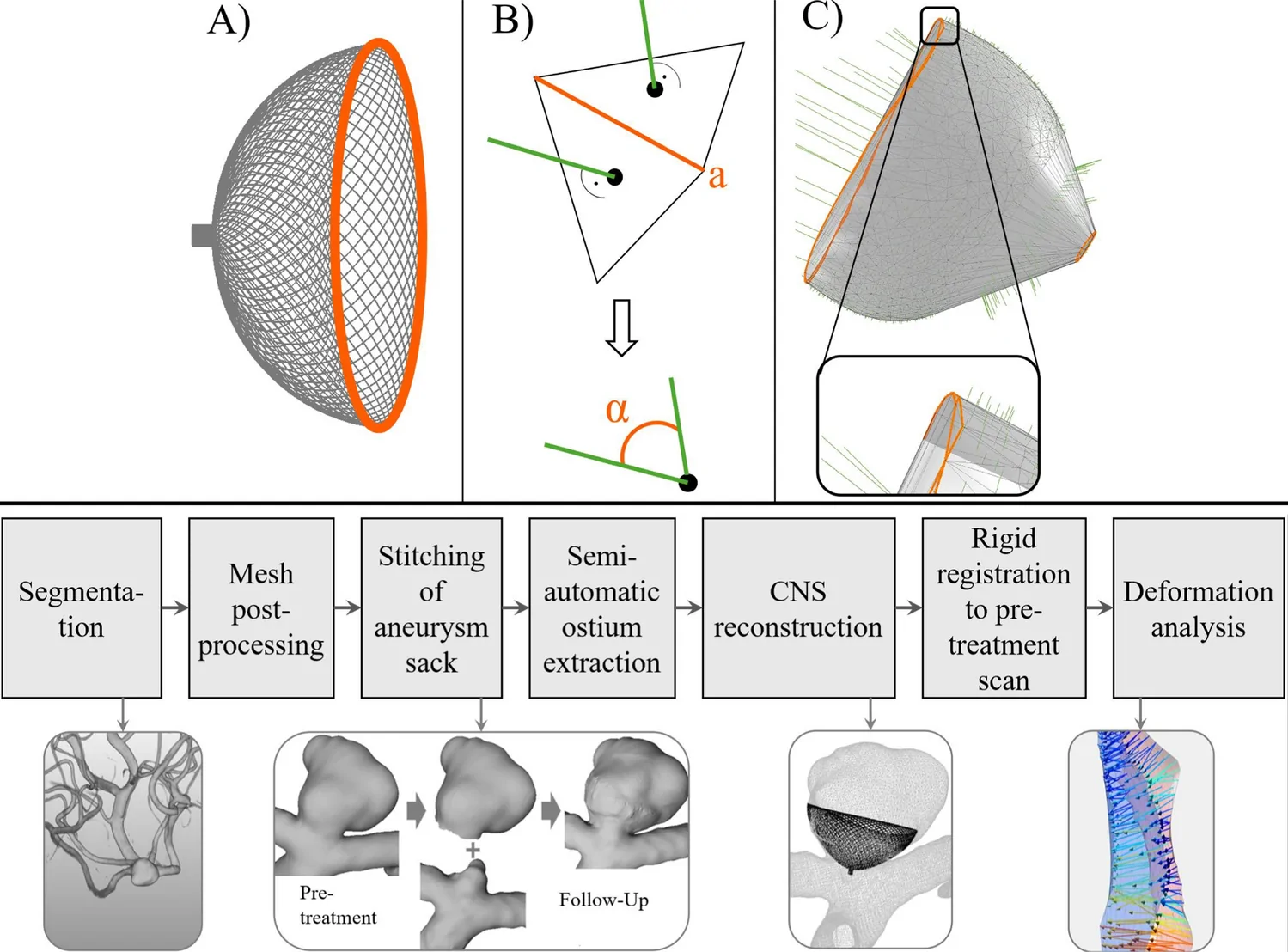
Upper: A) CNS with highlighted edge ring (orange). B) Angle α of edge a (orange), determined by face normals (green). C) CH of a CNS, with face normals (green) and candidate edges for edge ring (orange). Lower: Data processing pipeline, inspired by and expanding upon [13].

### Data Processing

We followed and expanded the data processing from [13], resulting in the pipeline in Fig. 2. An initial segmentation of the vasculature was adapted from [17], followed by manual artifact removal. To reduce modality-dependent variability, all datasets underwent the same segmentation, mesh reconstruction and post-processing workflow prior to analysis. Morphological parameters were extracted from triangulated surface meshes rather than directly from image intensities, thereby reducing the influence of modality-specific contrast characteristics.

As the IA sac was not visible in post-treatment scans due to the CNS disrupting flow of the contrast agent, the IA sac of the pre-treatment scan was manually stitched onto the neck remnant of the post-treatment IAs. The transition region between pre- and post-treatment meshes was defined outside the visible ostium contours present in both datasets to preserve the original ostium geometry. Care was taken to avoid artificial smoothing or reshaping, such that only the aneurysm dome was reconstructed while the ostium region remained unchanged. Consequently, only ostium-based parameters were included in the longitudinal morphological analysis.

#### Segmentation and Mesh Post-Processing

The CNS was coarsely segmented from the scans via the same methods. Due to the scan resolution, artifacts were present, and individual wires were not visible. A CAD model of the CNS, courtesy of Cerus Endovascular (acquired by Stryker in 2023), was then manually registered via the radiopaque markers, and was fitted via smooth deformation using a lattice modifier (Blender 3.6.4, The Blender Foundation, The Netherlands) to the CNS segmentation and into the vasculature.

#### Extraction of Morphology

IA ostium and pre-treatment IA morphological parameters were extracted semi-automatically as part of the segmentation pipeline.

Size and shape parameters of the ostia as well as the CNS were calculated in MATLAB R2023a (MathWorks, Natick, MA, USA), directly from triangulated surface meshes (vertices, faces, and edges). As the CNS is a complex double-layered wire mesh, we calculated size parameters based on its convex hull (CH) and its edge ring (see Fig. 2 A), meaning the top rim of the CNS. The triangulated mesh of the CNS convex hull was examined for edges where the connecting triangles had an angle >15$^\circ$. Angle was determined via the adjacent faces’ normals (see Fig. 2 B). Due to CNS shape, most edges had an angle close to 0$^\circ$, and only the radiopaque marker edge and edge ring of the CNS remained with less flat edge angles. However, the top ring edges were not perpendicular, necessitating a smaller threshold to capture a complete ring of candidate edges (see Fig. 2 C). These edges were connected to the edge ring.

#### Morphological Parameters

Size and shape of the CNS edge ring were assessed with the same parameters as the IA ostium: area, width (defined as maximum diameter), mean diameter, CH volume, and the shape indices ellipticity index (EI), non-circularity index (NCI), and undulation index (UI). For NCI and UI, 2D adaptations of the non-sphericity index and UI that do not require volume measurements were used [18].

For all parameters, yearly rate of change was calculated ($\frac{\Delta }{year}$) due to inconsistent intervals between scans.

All scans of one IA were rigidly co-registered using ICP via point-based alignment in Meshlab v2022.02 [19]. Registration was performed based on stable vascular structures of the parent vessels surrounding the aneurysm region after mesh preprocessing and segmentation cleanup. Alignment quality was visually verified in all cases by inspection of vessel overlap and anatomical consistency after co-registration. After registration, average symmetric surface distance (ASSD), the mean distance of all points of one mesh to all points on the other mesh and vice versa was calculated for ostium and CNS.

## Results

Using the co-registered meshes, we analyzed ASSD and morphological parameters of the ostium, CNS, and CNS edge ring. Parameters were grouped and compared based on scan timing and recurrence status. The groups were:

- **Group 0: Intra-patient variance.** Assesses deformation of scans taken on the same day or within a short interval without intervention, to determine the range of deformation caused by patient movement, segmentation ambiguity, resolution, etc. Acts as control group. Five patients: 02, 06, 07, 08, 10.
- **Group 1: Postprocedural changes.** Assesses deformation from pre- to postprocedural scans. Eight patients: 01, 02, 05, 06, 07, 09, 10, 12.
- **Group 2: Longitudinal changes.** Assesses deformation between pre-treatment and last follow-up scan. For CNS parameters, the postprocedural scan served as baseline (Scan 1), as the CNS is only visible after deployment. All 13 patients.
- **Group 3: Changes over 6 months versus after more than one year.** Assesses differences in rates of change within the first 6 months (6 m) and after more than one year (1y+). 6 m: Eight patients: 01, 02, 05, 06, 07, 09, 11, 12. 1y+: Nine patients: 01, 02, 04, 05, 06, 07, 09, 11, 13.
- **Group 4: Correlation with recurrence.** Assesses correlation between initial morphology and recurrence, and morphological differences of IAs with and without recurrence, as well as correlation of morphological changes over time. All 13 patients.
- **Group 5: Spatial changes.** Assesses positional displacement of the CNS via ASSD. All 13 patients.

### Statistical Analysis

For statistical analysis, we used RStudio 2024.04.2 (Posit Software, PBC, USA). A paired Student’s t-test was performed for pairwise comparison. Normality was assessed with Shapiro-Wilk’s method, as well as visually inspected using QQ-plots. Homogeneity of variance was assessed with the Levene test. In case of non-normality or inhomogeneity, a Mann–Whitney-Wilcoxon test was used. To quantify the risk of a Type II error given our sample size, we performed a post-hoc power analysis for all morphological parameters and all evaluated groups. Statistical power and effect sizes (Cohen’s d) were estimated, allowing us to evaluate the likelihood of detecting true differences given the sample size and variability in our dataset.

### Group 0: Intra-Patient Variance

Group 0 assesses the range of observed deformation due to patient movement, different scanning devices, segmentation ambiguity, scan resolution, etc. The terms “shrinking” and “increase” refer to the range of intra-patient variance and deformation that can occur due to the mentioned reasons.

The range of relevant parameter changes was as follows:

- Ostium area Δ: −8.99 to 4.56 mm^2^
- Ostium width Δ: −1.87 to 0.74 mm
- Ostium mean diameter Δ: −1.27 to 0.56 mm
- Ostium EI Δ: −0.22 to 0.11
- Ostium UI Δ: −0.04 to 0.04
- Ostium NCI Δ: −0.11 to 0.06

Two pairs of scans featuring a CNS were taken on the same day. One CNS grew by 23.65 mm^2^, the other shrank by 18.05 mm^2^. This opposite trend between the two patients (one increasing, one decreasing) was consistent across all size parameters. For CNS ring area, one case grew by 2.02 mm^2^, and the other decreased by −10.89 mm^2^. Shape parameters UI and NCI of the CNS ring showed no change, while both scans showed a small ring EI increase.

No statistically significant changes were observed in the intra-patient variance group (all Δ p ≥ 0.05). Effect sizes (Cohen’s d) were small (d < 0.35) and statistical power was very low (< 0.1) (Table 1).

**Table 1:** Average results per parameter of scan 1 and scan 2 of group 0, including standard deviation, absolute change, Cohen’s d and Power.

Group 0	Scan 1	Scan 2	Δ	Cohen’s d	Power
*Ostium Parameters (n = 5)*					
Area	24.02 ± 8.92	25.15 ± 10.25	1.14 ± 5.20	0.20	0.06
Width	6.56 ± 1.41	6.40 ± 1.24	−0.16 ± 0.92	−0.15	0.06
Mean Dia	5.38 ± 1.05	5.46 ± 1.16	0.07 ± 0.69	0.10	0.05
CH Vol	14.89 ± 6.36	15.88 ± 3.45	0.99 ± 5.81	0.15	0.06
EI	0.57 ± 0.07	0.52 ± 0.09	−0.04 ± 0.11	−0.34	0.09
UI	0.55 ± 0.01	0.54 ± 0.01	0.00 ± 0.02	−0.14	0.06
NCI	0.14 ± 0.03	0.12 ± 0.04	−0.02 ± 0.06	−0.27	0.08
*CNS Parameters (n = 2)*					
Area	111.10 ± 42.66	113.90 ± 21.81	2.80 ± 20.37		
CH Vol	87.05 ± 49.52	86.50 ± 26.23	−0.55 ± 23.29		
Ring area	30.45 ± 13.87	26.01 ± 7.41	−4.44 ± 6.64		
Ring width	6.84 ± 1.48	5.47 ± 0.86	−0.38 ± 0.61		
Ring mean Dia	6.16 ± 1.41	5.80 ± 0.85	−0.36 ± 0.57		
Ring EI	0.59 ± 0.01	0.68 ± 0.02	0.09 ± 0.01		
Ring UI	0.50 ± 0.00	0.50 ± 0.00	0.00 ± 0.00		
Ring NCI	0.02 ± 0.01	0.02 ± 0.00	0.00 ± 0.00		

The latter two were not calculated for CNS Parameters, as there were only two cases. Vol stands for volume, Dia for diameter. * marks statistical significance

### Group 1: Postprocedural Changes

Ostium size increased after CNS deployment, although changes were not statistically significant (Table 2). EI decreased and showed the widest variation of all shape indices (0.46 to 0.73). UI stayed between 0.52 and 0.57. NCI stayed low, ranging between 0.05 and 0.17.

Significant changes were observed in:

- Ostium EI: $\Delta = -0.07 \pm 0.06$, p-value = 0.01, d=-1.18, power = 0.82
- Ostium NCI: $\Delta = +0.04 \pm 0.04$, p-value = 0.04, d=0.90, power = 0.60

Other parameters showed no significant changes (p-value ≥ 0.05), with low power (< 0.35), with the exception of Ostium UI, which had a large effect size (d=0.81) but p-value ≥ 0.05.

**Table 2:** Average results per parameter of scan 1 and scan 2 of group 1, including standard deviation, absolute change, Cohen’s d and Power.

Group 1	Scan 1	Scan 2	Δ	Cohen’s d	Power
*Ostium Parameters (n = 8)*					
Area	20.75 ± 9.76	22.04 ± 10.29	1.29 ± 4.77	0.25	0.10
Width	5.79 ± 1.30	6.03 ± 1.32	0.24 ± 0.91	0.25	0.09
Mean Dia	5.01 ± 1.08	5.06 ± 1.18	0.05 ± 0.55	0.09	0.06
CH Vol	10.45 ± 6.00	15.45 ± 8.92	5.00 ± 7.74	0.63	0.34
EI	0.59 ± 0.08	0.51 ± 0.07	−0.07 ± 0.06*	−1.18*	0.82*
UI	0.53 ± 0.01	0.55 ± 0.02	0.01 ± 0.01	0.81*	0.51
NCI	0.10 ± 0.03	0.13 ± 0.04	0.04 ± 0.04*	0.90*	0.60

Vol stands for volume, Dia for diameter. * marks statistical significance

### Group 2: Longitudinal Changes

**Fig. 3:**
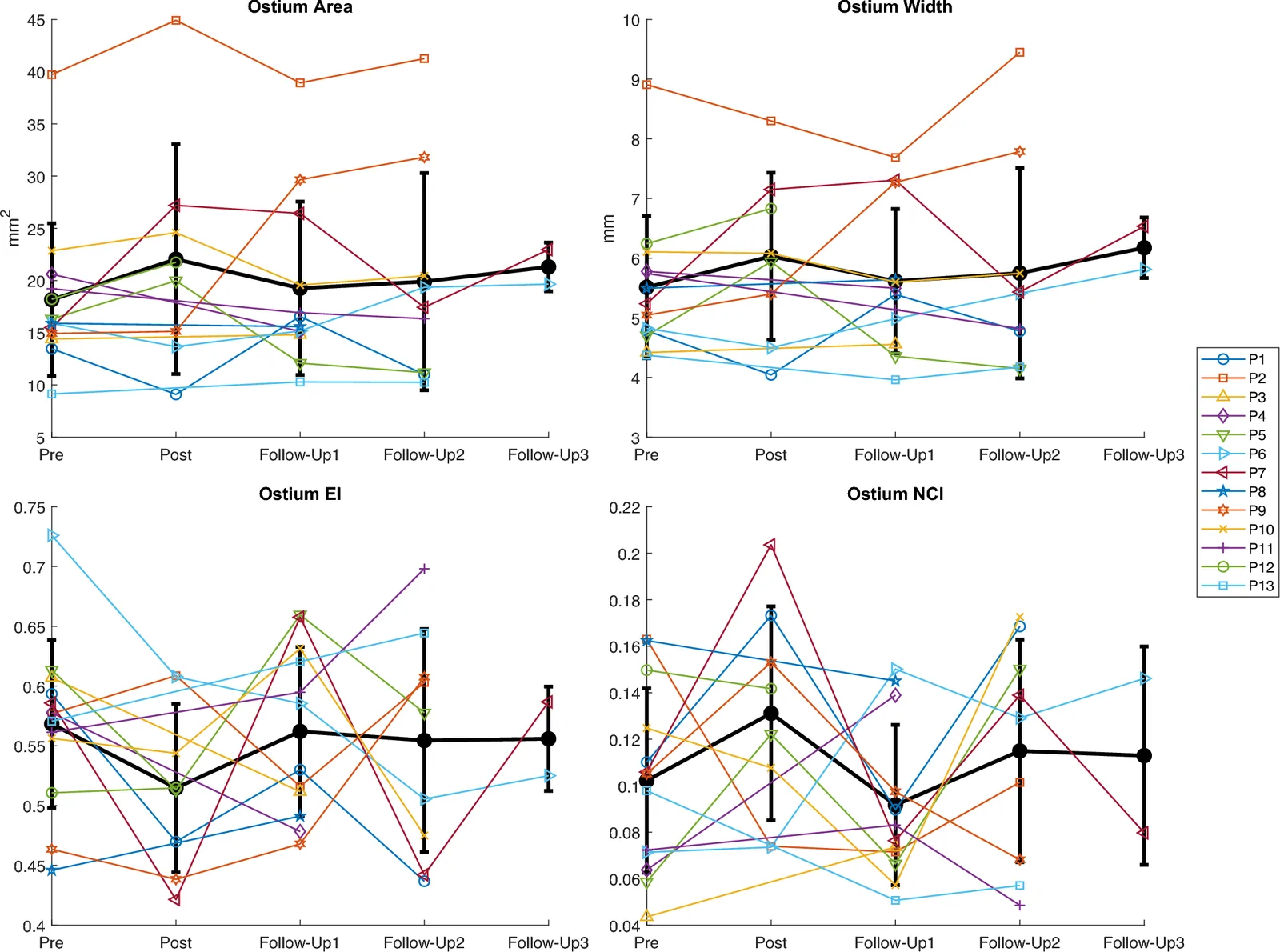
Development of selected ostium parameters over the scans for each patient. Thin colored lines indicate individual patients. Black line and error bars represent mean ± standard deviation.

Ostium size changed very little over time (Table 3). More ostia decreased in size than increased, but one patient showed a particularly strong increase in ostium size, ostium area alone increasing by a total of 16.89 mm^2^ (Fig. 3, P9), while total area change of all other ostia ranged between ±0.41 and ±7.46 mm^2^. The same patient increased by 2.74 mm in width and 2.19 mm in mean diameter, where the rest of the IAs width and mean diameter change ranged from ± 0.01 to 1.39 mm and ±0.01 to 1.03 mm, respectively. Excluding this patient would result in mean changes of −2.20 ±4.15 $\frac{mm^{2}}{year}$ for area, −0.15 ±1.00 $\frac{mm}{year}$ for width, and −0.24 ±0.55 $\frac{mm}{year}$ for mean diameter. Changes of ostium shape indices were small.

**Fig. 4:**
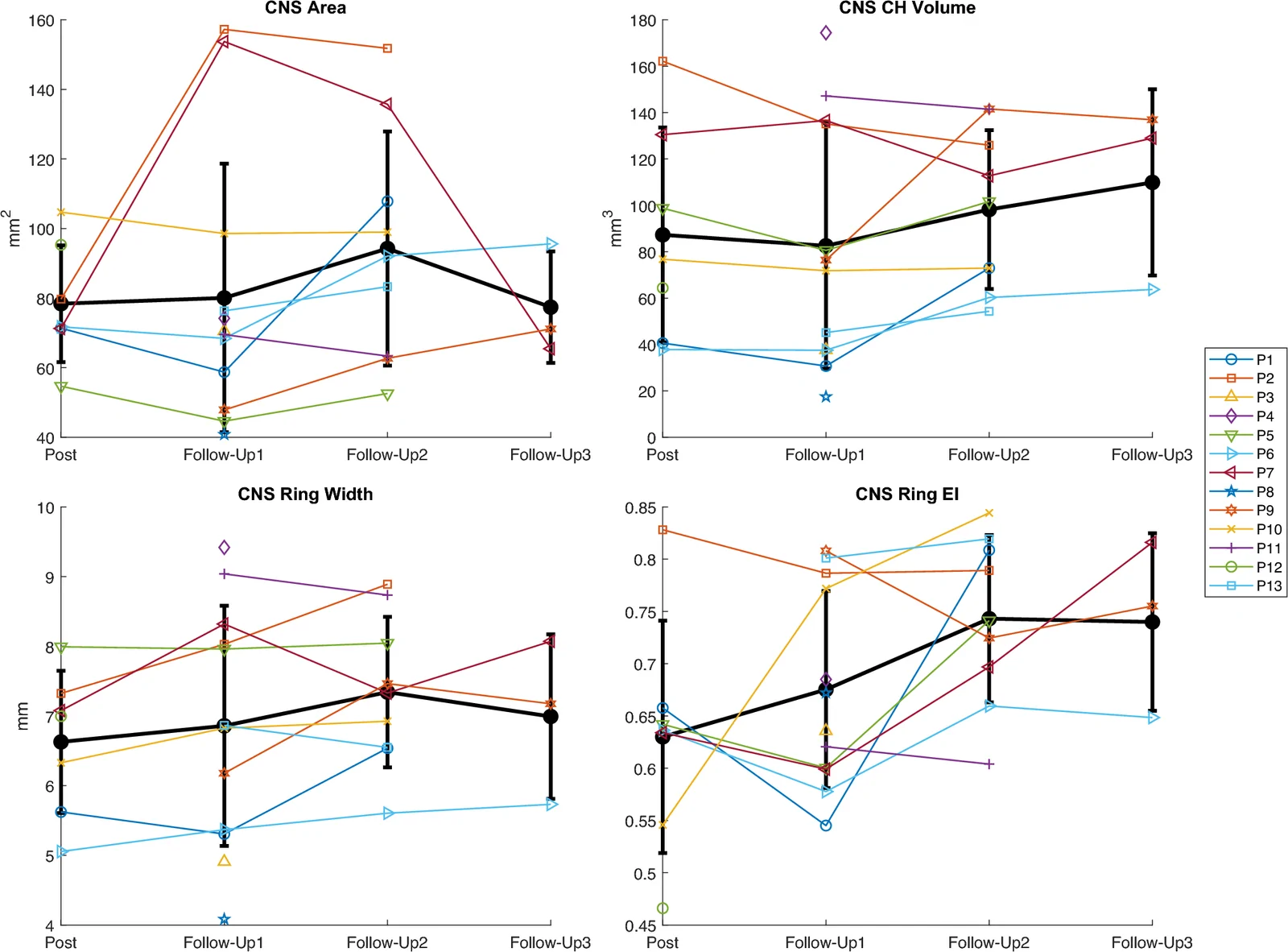
Development of selected CNS parameters over the scans for each patient. Thin colored lines indicate individual patients. Black line and error bars represent mean ± standard deviation.

The CNS and its edge ring size increased over time on average. Half of the ten patients with more than one CNS scan showed an increase in CNS area (Fig. 4). Those whose area decreased, however, shrank by a range of −2.10 to −14.07 mm^2^, while the range of increase of those whose area grew was 6.95 to 72.07 mm^2^. The CNS edge ring only decreased in three of the ten patients. The CNS edge ring changed little in shape.

No statistically significant changes were observed for any Ostium or CNS longitudinal changes (Table 3). Some moderate effect sizes were noted (Cohen’s d ≈ 0.48-−0.73), but power remained low (< 0.55).

**Table 3:** Average results per parameter of scan 1 and scan 2 of group 2, including standard deviation, absolute change, Cohen’s d and Power. Vol stands for volume, Dia for diameter. * marks statistical significance

Group 2	Scan 1	Scan 2	Δ/year	Cohen’s d	Power
*Ostium Parameters (n = 13)*					
Area	18.97 ± 8.37	18.78 ± 8.58	−1.09 ± 6.07	0.07	0.06
Width	5.56 ± 1.13	5.68 ± 1.46	0.03 ± 1.20	0.26	0.14
Mean dia	4.76 ± 0.96	4.73 ± 1.04	−0.10 ± 0.79	0.08	0.06
CH Vol	9.65 ± 5.28	10.52 ± 6.51	0.08 ± 6.60	0.36	0.23
EI	0.58 ± 0.07	0.56 ± 0.08	−0.01 ± 0.12	−0.24	0.13
UI	0.53 ± 0.01	0.54 ± 0.02	0.01 ± 0.02	0.46	0.33
NCI	0.09 ± 0.04	0.11 ± 0.04	0.01 ± 0.06	0.46	0.33
*CNS Parameters (n = 10)*					
Area	74.25 ± 15.91	87.12 ± 27.12	9.75 ± 26.50	0.49	0.28
CH vol	87.98 ± 42.83	94.71 ± 34.44	1.96 ± 24.56	0.25	0.11
Ring area	31.29 ± 9.55	35.53 ± 11.30	4.69 ± 6.64	0.72	0.52
Ring width	6.85 ± 1.09	7.28 ± 1.04	0.30 ± 0.86	0.56	0.36
Ring mean dia	6.29 ± 0.98	6.78 ± 1.05	0.40 ± 0.62	0.73	0.54
Ring EI	0.66 ± 0.11	0.75 ± 0.08	0.10 ± 0.17	0.72	0.53
Ring UI	0.50 ± 0.00	0.50 ± 0.01	0.00 ± 0.00	−0.48	0.28
Ring NCI	0.01 ± 0.01	0.01 ± 0.00	0.00 ± 0.00	−0.27	0.12

### Group 3: Changes Over Six Months Versus After Years

For most parameters, the 6 m $\frac{\Delta }{year}$ was larger than the 1y+ $\frac{\Delta }{year}$, except for ostium CH volume, NCI, CNS width, and CNS ring EI.

CNS Volume showed a statistically significant change (p-value = 0.02), an average decrease of −19.61 ±24.36 $\frac{mm^{3}}{year}$ at 6 m, and then an average increase of 20.52 ± 34.05 $\frac{mm^{3}}{year}$ at 1y+. While effect size was high with Cohen’s d =-1.32, power was low with power =0.61. Other parameters were not significant, with small to moderate effect sizes and low power (Table 4).

**Table 4:** Average yearly change per parameter (Δ/year) of scans after 6 months (6 m) and scans after over a year (1y+), including standard deviation, Cohen’s d and Power.

Group 3	6 m Δ/year	1y+ Δ/year	Cohen’s d	Power
*Ostium parameters*				
Area	1.81 ± 14.22	0.66 ± 3.50	−0.10	0.05
Width	0.31 ± 2.20	0.62 ± 1.27	−0.41	0.12
Mean dia	0.53 ± 1.81	0.13 ± 0.55	0.02	0.05
CH Vol	−1.88 ± 20.55	−3.82 ± 5.79	−0.02	0.05
EI	0.12 ± 0.21	0.05 ± 0.11	0.49	0.16
UI	−0.02 ± 0.05	0.00 ± 0.02	−0.61	0.21
NCI	−0.07 ± 0.13	0.02 ± 0.05	−0.93*	0.44
*CNS parameters*				
Area	57.97 ± 92.94	−2.33 ± 34.00	0.66	0.20
CH Vol	−19.61 ± 24.36	20.52 ± 34.05*	−1.32*	0.61
Ring area	9.94 ± 8.93	7.89 ± 8.98	−0.02	0.05
Ring width	0.82 ± 1.20	0.71 ± 0.81	−0.26	0.07
Ring mean Dia	0.91 ± 0.86	0.72 ± 0.77	−0.09	0.05
Ring EI	−0.12 ± 0.06	0.05 ± 0.11	−0.35	0.09
Ring UI	0.00 ± 0.00	0.00 ± 0.00	0.08	0.05
Ring NCI	0.00 ± 0.01	0.00 ± 0.00	−0.71	0.23

Vol stands for volume, Dia for diameter. * marks statistical significance

### Group 4: Correlation with Recurrence

Looking at pre-treatment parameters of the IAs (including the IA sac), one of the cases with recurrence was a small IA (area: 43.61 mm^2^, width: 5.27 mm, CH volume: 37.90 mm^3^, AR: 0.66). Another case was a large aneurysm (area: 309.21 mm^2^, width: 12.74 mm, CH volume: 488.02 mm^3^, AR: 3.06). That first IA is the smallest IA of all cases, while there was one case with full occlusion that was the largest IA (area: 316.36 mm^2^, width: 13.62 mm, CH volume: 543.12 mm^3^, AR: 2.56). There was no statistically significant correlation between recurrence and any morphological pre-treatment IA parameter. Differences between recurrence and full occlusion cases were also non-significant, with low effect sizes and power (Table 5).

In group 2, longitudinal change, two parameters had statistically significant differences between recurrence and full occlusion cases: $\frac{\Delta }{year}$ of ostium EI (p-value: 0.02) and of ostium NCI (p-value: 0.03).

Ostium EI change for recurrence cases was −0.05 ±0.04 $\frac{\Delta }{year}$, and 0.08 ±0.08 $\frac{\Delta }{year}$ for full occlusion cases. Of the recurrence cases, two cases had a decrease in EI of - 0.08 and −0.07, while the last had a small increase of 0.01. From the full occlusion cases, only two had a decrease in EI, both being very small, −0.02 and −0.01. Change of ostium NCI was 0.05 ±0.03 $\frac{\Delta }{year}$ for recurrence cases and −0.03 ±0.05 $\frac{\Delta }{year}$ for full occlusion cases. All three recurrence cases’ NCI increased, while of the full occlusion cases all but two showed an NCI decrease.

**Table 5:** Average results per IA parameters before treatment for all patients, patients with full occlusion, and with reperfusion after treatment. Including standard deviation, Cohen’s d and Power.

Group 4	All	Full occlusion	Reperfusion	Cohen’s d	Power
	(n = 12)	(n = 9)	(n = 3)		
*IA Parameters*					
Area	168.29 ± 86.91	166.54 ± 78.33	173.54 ± 108.51	0.40	0.08
Width	9.39 ± 2.31	9.47 ± 2.00	9.15 ± 3.06	0.13	0.05
CH Vol	244.20 ± 162.12	240.70 ± 153.93	254.70 ± 184.13	−0.08	0.05
Aspect Ratio	1.57 ± 0.68	1.53 ± 0.52	1.69 ± 1.01	−0.22	0.06
EI	0.25 ± 0.01	0.25 ± 0.01	0.26 ± 0.02	−0.56	0.12
UI	0.09 ± 0.04	0.10 ± 0.04	0.07 ± 0.002	0.60	0.13
NSI	0.18 ± 0.05	0.19 ± 0.03	0.16 ± 0.11	0.60	0.13

Vol stands for volume, Dia for diameter. * marks statistical significance

### Group 5: Spatial Changes

**Fig. 5:**
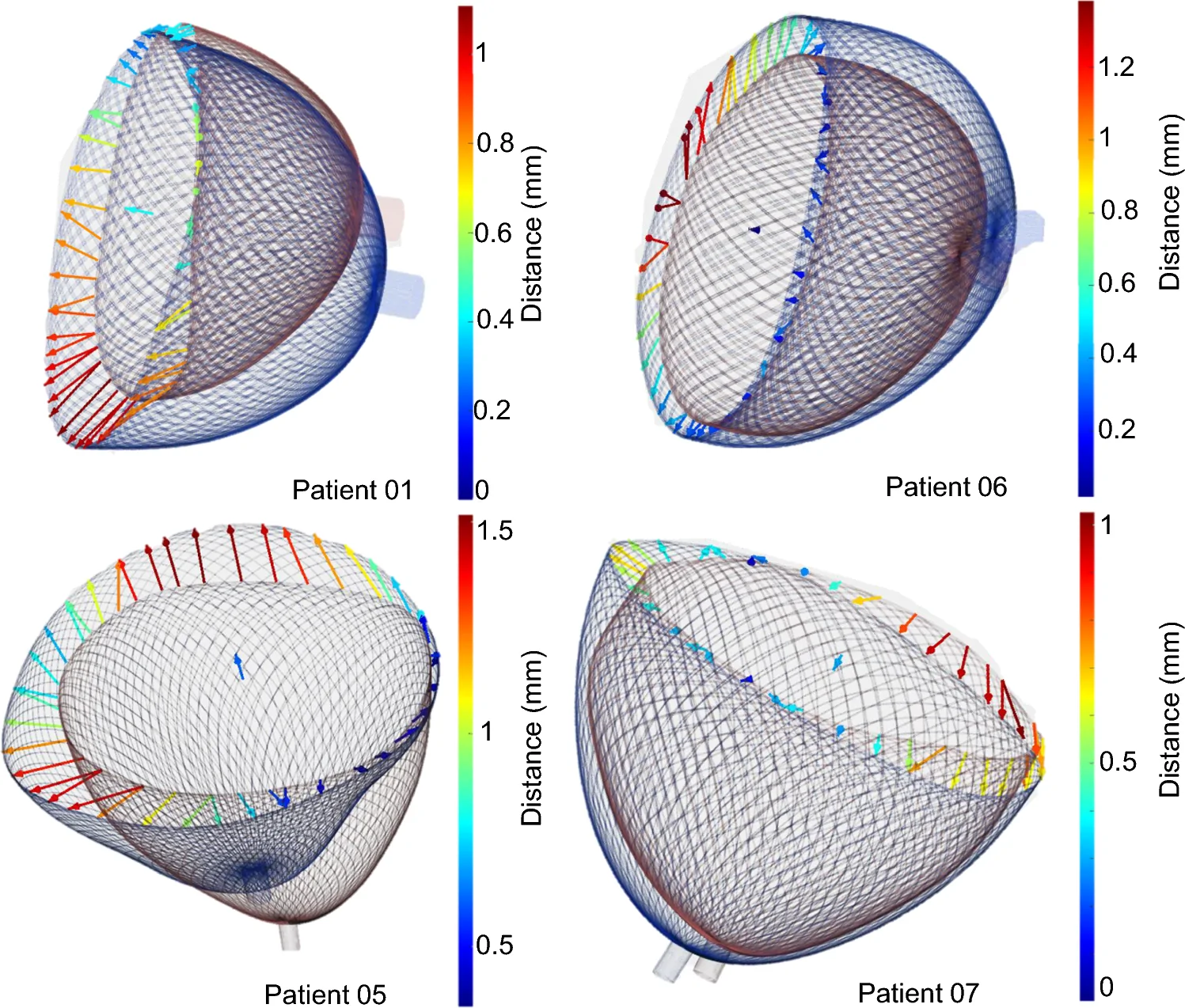
Example cases of CNS, registered and with color-coded arrows highlighting deformation at the edge ring. Red CNS are postprocedural, blue CNS are latest follow-up.

Ostia had an average ASSD of 0.54 ±0.29 mm and the CNS had an average ASSD of 0.63 ±0.33 mm. ASSD of group 0, intra-patient variance, was 0.55 ±0.33 mm for ostia, and 0.47 ±0.23 mm for CNS. For group 2, longitudinal change, ASSD was 0.55 ±0.26 mm on average for ostia, and 0.64 ±0.35 mm for CNS for all patients. The three IAs with a recurrence had an average ASSD of 0.44 ±0.27 for ostia, and 0.55 ±0.33 mm for CNS, while the ten IAs with full occlusion had an average ASSD of 0.58 ±0.25 mm for ostia and 0.69 ±0.36 mm for CNS (Fig. 5).

One recurrence case had a low ASSD of 0.25 ±0.12 mm, and the other two cases with recurrence also had below-average ASSD. Overall, ostia ASSD ranged between 0.23 and 0.88 mm. The case with smallest ostium ASSD was a case with full occlusion with ostium ASSD 0.23 ±0.10 mm. One full occlusion case had a high ASSD of 1.39 ±0.89 mm, while the rest of CNS ASSD ranged from 0.35 to 0.74 mm. The cases with both the highest and lowest CNS ASSD were full occlusion cases.

We found no statistically significant spatial changes.

## Discussion

We compared a variety of morphological parameters of IAs treated with the CNS over multiple follow-up scans.

### Comparison to Intra-Patient Variance

Group 0, intra-patient variance, was generally smaller than postprocedural changes (group 1). The ranges were comparable except for ostium CH volume, which was 5.00 ±7.74 mm^3^ in group 1 and 0.99 ±5.81 mm^3^ in group 0. There was no statistically significant difference between the groups.

CNS deformation of group 0 and group 2 was very different, developing in different directions for CNS CH volume, CNS ring area, CNS ring width, and mean diameter. The absolute change was generally bigger in group 2, and a limitation of group 0 is the small number of cases with CNS (n = 2), which markedly also deformed in different directions. Group 2 deformations were overall within observed intra-patient variance.

The magnitude of the measured differences Δ between group 0 and all other groups were not statistically significant, suggesting that some observed short-term differences may reflect measurement noise.

Combined with the lack of correlation between time and recurrence and any of the parameters, no measurable effect of CNS deployment on ostium morphology was detected. This suggests possible robustness of the CNS over time, however, lack of statistical significance does not mean a lack of deformation, so a study to confirm this is necessary. These findings suggest that due to the lack of statistical significance or observed changes between recurrence and full occlusion cases, recurrence cannot be predicted by IA morphology. While this result is in line with another recent study [4], given the small sample size and limited power, this conclusion should be interpreted with caution.

### Comparison of Observations

The strongest deformations occurred in group 1, postprocedural changes.

The statistical significance, large effect sizes and adequate power of postprocedural changes of ostium EI and NCI suggest a measurable deformation of the ostium after CNS deployment. EI decreased on average and in six out of eight cases. The two other cases showed a very small increase (<0.01) and were also the only cases where NCI decreased. Most aneurysms had a less elliptical ostium after CNS deployment, while the ostium was also less perfectly circular.

Group 1’s changes are stronger than group 2’s. The direction of the deformation was the same for all parameters except for ostium area. This may be due to an initial postprocedural deformation that leads to an increase in area, whereas over time, the healing process facilitated by the CNS could lead to a decrease in area. However, neither change was statistically significant.

In group 3, 6 m and 1y+ changes, we found that 6 m $\frac{\Delta }{year}$ is almost always stronger than 1y+ $\frac{\Delta }{year}$, indicating that the strongest changes happen in the time after deployment with the CNS settling and becoming more stable the more time passes. We, however, only observed statistically significant changes when comparing 6 m and 1y+ $\frac{\Delta }{year}$ for CNS CH volume. CNS CH volume shrinks and then grows 1y+ later. The growing in the year later may be due to thrombus formation expanding the visible CNS. Statistical power, however, was generally low, indicating that potential long-term morphological changes were either small or obscured by measurement variability.

### Comparison to Preliminary Study

A preliminary study [13] performed a longitudinal comparison of seven ostia with one pre-interventional scan and one follow-up scan. In group 2, longitudinal change, we found similar ostium area shrinkage over time, namely a yearly shrinkage of −1.09 ±6.07 $\frac{mm^{2}}{year}$, compared to the preliminary study’s −0.58 ± 4.88 $\frac{mm^{2}}{year}$. Here, however, an increase of ostium width was found, though, upon exclusion of a case with a particularly high ostium size increase after treatment, average ostium width decreases to −0.15 ±1.00 $\frac{mm}{year}$, compared to −0.59 ±0.87 $\frac{mm}{year}$ in the preliminary study. Mean diameter decrease in our longitudinal analysis was comparable to the preliminary study’s. Our results for shape changes are comparable with the preliminary study.

Unlike in the preliminary study, here we found no correlation or statistical significance between time passing and ostium area shrinkage, or any other parameters.

### Comparison to Other Device Deformations

Other IFDs are subject to deformations. For example, the Woven EndoBridge (WEB), the major alternative of the CNS, the compression phenomenon is discussed, where the entire device seems to shrink within the IA sac. Median shrinkage is about 50% of WEB height [14]. Exact causes of the deformation are unclear, but studies report this occurring in 25 to 57.2% of cases [20]. The compression seems to not affect occlusion rate [14, 20].

Deformations have also been reported for stents, affecting the struts of the stent, porosity and pore density, altering blood flow through the stent after deployment [16]. Another concern is vessel straightening, where the radius of curvature of the parent vessel increases 3.6 ±2.7 mm after stent deployment, which is more than 50% of the pre-deployment value [15].

In contrast, the changes we observed in CNS deformation were smaller and not statistically significant. The ASSD remained below 1 mm, which is markedly lower than the deformation magnitudes reported for the WEB and stents. While direct quantitative comparison is limited due to differences in devices, study design, and evaluated endpoints, the substantially smaller deformation metrics and negligible effect sizes observed in this study indicate a higher geometric stability of the CNS over time. The absence of statistically significant differences was accompanied by small effect sizes, further supporting that the observed geometric changes are minor compared to deformation phenomena reported in other devices. This indicates the robustness of the device and suggests that deformation-related flow alterations may be less pronounced compared to previously reported IFDs.

### Limitations

The observed changes may partially be due to modality, scan resolution and segmentation ambiguity. The retrospective multicenter dataset included both 3D rotational DSA and 3D CTA acquisitions across timepoints, which may introduce modality-dependent variability related to reconstruction characteristics, contrast behavior, and metal artifacts. Scan resolution ranged from 0.16 to 0.47 mm, exceeding the magnitude of some observed changes. Additionally, scans are subject to noise. While segmentation was performed as consistently as possible by an experienced user, there is ambiguity during the segmentation process. Ambiguities arise not only from image noise but also from limited contrast resolution, partial volume effects, vessel overlap, and locally reduced signal near the implant surface, which can affect boundary delineation. The same case may have a range of segmentation parameters that may appear as a valid segmentation, without a definite ground truth. For CNS segmentation, overexposure of the radio-opaque marker posed an additional challenge. This, along with patient movement as well as the brain not being a static object, may partially be the reason for the observed deformations.

Recent deep learning studies have demonstrated high performance for automatic aneurysm segmentation in angiography and CT datasets, achieving Dice scores around 0.9 and enabling more consistent morphological measurements [21–24]. However, such success often depends on large annotated datasets that are currently unavailable for CT and DSA with CNS implants. While automatic segmentation can reduce observer variability and improve robustness, it is unlikely to fully eliminate ambiguity caused by artifact susceptibility and limited imaging data.

Deformation varied strongly from patient to patient, as did the time between follow-ups. 13 aneurysms with follow-ups ranging from six to 26 months may not be able to represent every possible deformation case, and as our power analysis indicated, a substantially larger sample size is required to reliably detect moderate effects (Cohen’s d ≈ 0.5) with power ≥ 0.8 to confirm our results. This may also be why the study resulted in a lack of statistically significant results, low power, and low effect sizes. While no measurable significant change after intervention may seem promising, a lack of statistically significant deformation does not mean there is no deformation. Future studies should include larger, multicenter cohorts, longitudinal follow-up data over a longer time-frame, and combined experimental and computational validation to further assess the clinical relevance and robustness of the CNS.

## Conclusion

In this study, we analyzed morphological changes of IAs and the CNS over multiple follow-up scans using co-registered 3D meshes reconstructed from 3D DSA and CTA scans. Among all evaluated parameters, only immediate postprocedural changes in the ostium’s EI and NCI reached statistical significance, accompanied by large effect sizes and sufficient statistical power. These findings suggest that CNS deployment induces measurable short-term shape changes at the ostium.

No statistically significant long-term morphological changes of either the ostium or CNS were detected across follow-up periods up to 26 months. Similarly, no correlation was observed between recurrence and morphological parameters, nor between morphological deformation and time after treatment. However, due to the small sample size, heterogeneous follow-up intervals, and limited imaging resolution, where several measured deformations were smaller than the voxel size, these results must be interpreted with caution. Small or subtle changes cannot be excluded.

Overall, our findings suggest that the CNS remains morphologically stable over time and does not induce measurable deformation of the aneurysm. This stability may contribute to the device’s efficacy and durability. Nevertheless, further studies with larger patient cohorts, standardized follow-up imaging, and higher-resolution modalities are required to confirm these observations and to more precisely quantify potential morphological changes.
